# Mechanical Recycling of Partially Bio-Based and Recycled Polyethylene Terephthalate Blends by Reactive Extrusion with Poly(styrene-*co*-glycidyl methacrylate)

**DOI:** 10.3390/polym12010174

**Published:** 2020-01-09

**Authors:** Sergi Montava-Jorda, Diego Lascano, Luis Quiles-Carrillo, Nestor Montanes, Teodomiro Boronat, Antonio Vicente Martinez-Sanz, Santiago Ferrandiz-Bou, Sergio Torres-Giner

**Affiliations:** 1 Department of Mechanical and Materials Engineering (DIMM), Universitat Politècnica de València (UPV), Plaza Ferrándiz y Carbonell 1, 03801 Alcoy, Spain; sermonjo@mcm.upv.es; 2 Technological Institute of Materials (ITM), Universitat Politècnica de València (UPV), Plaza Ferrándiz y Carbonell 1, 03801 Alcoy, Spain; dielas@epsa.upv.es (D.L.); luiquic1@epsa.upv.es (L.Q.-C.); nesmonmu@upvnet.upv.es (N.M.); tboronat@dimm.upv.es (T.B.); sferrand@mcm.upv.es (S.F.-B.); 3 Escuela Politécnica Nacional, Ladrón de Guevara E11-253, Quito 17-01-2759, Ecuador; 4 Institute of Design and Manufacturing (IDF), Universitat Politècnica de València (UPV), Plaza Ferrándiz y Carbonell 1, 03801 Alcoy, Spain; anmarsan@mcm.upv.es; 5 Novel Materials and Nanotechnology Group, Institute of Agrochemistry and Food Technology (IATA), Spanish National Research Council (CSIC), Calle Catedrático Agustín Escardino Benlloch 7, 46980 Paterna, Spain

**Keywords:** bio-PET, r-PET, chain extenders, reactive extrusion, secondary recycling, food packaging

## Abstract

In the present study, partially bio-based polyethylene terephthalate (bio-PET) was melt-mixed at 15–45 wt% with recycled polyethylene terephthalate (r-PET) obtained from remnants of the injection blowing process of contaminant-free food-use bottles. The resultant compounded materials were thereafter shaped into pieces by injection molding for characterization. Poly(styrene-*co*-glycidyl methacrylate) (PS-*co*-GMA) was added at 1–5 parts per hundred resin (phr) of polyester blend during the extrusion process to counteract the ductility and toughness reduction that occurred in the bio-PET pieces after the incorporation of r-PET. This random copolymer effectively acted as a chain extender in the polyester blend, resulting in injection-molded pieces with slightly higher mechanical resistance properties and nearly the same ductility and toughness than those of neat bio-PET. In particular, for the polyester blend containing 45 wt% of r-PET, elongation at break (ε_b_) increased from 10.8% to 378.8% after the addition of 5 phr of PS-*co*-GMA, while impact strength also improved from 1.84 kJ·m^−2^ to 2.52 kJ·m^−2^. The mechanical enhancement attained was related to the formation of branched and larger macromolecules by a mechanism of chain extension based on the reaction of the multiple glycidyl methacrylate (GMA) groups present in PS-*co*-GMA with the hydroxyl (–OH) and carboxyl (–COOH) terminal groups of both bio-PET and r-PET. Furthermore, all the polyester blend pieces showed thermal and dimensional stabilities similar to those of neat bio-PET, remaining stable up to more than 400 °C. Therefore, the use low contents of the tested multi-functional copolymer can successfully restore the properties of bio-based but non-biodegradable polyesters during melt reprocessing with their recycled petrochemical counterparts and an effective mechanical recycling is achieved.

## 1. Introduction

The transition of the plastic industry from its traditional Linear Economy to a Circular Economy, a more valuable and sustainable model, is being spearheaded by the European Union (EU), where legislative measures are being introduced to eliminate excessive waste [1]. To this end, it is first necessary to promote sustainable polymer technologies that decouple plastics from fossil feedstocks [2]. Furthermore, it is also important to increase the quality and uptake of plastic recycling [3]. In this context, the food packaging sector it is among the most heavily scrutinized, given the large production and the short life cycle of their products [4]. Polyethylene terephthalate (PET) is a thermoplastic polyester that is widely used in the manufacture of bottles for water or beverages and also food trays due to its good mechanical properties, chemical resistance, clarity, thermal stability, barrier properties, and low production cost [5]. PET is fully recyclable and it is, indeed, one of the most recycled plastics in the world. Since 2012, PET monomaterial packaging has showed recycling rates of approximately 52% in the EU and 31% in the United States (US) [6]. Moreover, the recycling of PET articles is expected to increase, as exemplified by companies like Coca-Cola, which have already announced a full switch to recycled plastics for their beverage bottles in some EU countries, starting with at least 50% rates by the end of 2023 [7].

In recent years, the replacement of conventional or petrochemical polymer materials with those obtained from natural and renewable sources is of great interest for research due to the depletion of fossil resources and the cost of extracting them. The main interest and advantage of using bio-based polymers reflects the concept of the so-called “biorefinery system design” due to its ability to improve the environmental impact of a product by reducing greenhouse gas emissions, economizing fossil resources, exploring the possibility of using a local resource, and the use of by-products or even wastes [8]. Bio-based polymers, which can be either biodegradable or non-biodegradable, are certified according to international standards such as EN 16640:2015, ISO 16620-4:2016, ASTM 6866-18, and EN 16785-1:2015. In particular, EN 16640:2015, ISO 16620-4:2016, and ASTM 6866-18 measure the bio-based carbon content in a material through 14C measurements, while EN 16785-1:2015 measures the bio-based content of a material using radiocarbon and elemental analyses [9]. Indeed, the pattern of production of biopolymers is shifting from biodegradable to bio-based. Bio-based but non-biodegradable polymers represented 57.1% of the total biopolymer production in 2018, whereas biodegradable polymers accounted for 42.9% [10]. This is in contradiction to the public perception that most biopolymers are biodegradable. Among them, partially bio-based polyethylene terephthalate (bio-PET) is currently the most produced bioplastic, reaching ~27% of the total production in 2018, that is, 0.54 million tons per year. [11]. This is based on the fact that this “green polyester” offers an almost identical chemical structure and properties to its petrochemical counterpart, that is, PET.

Several studies on PET recycling methods have indicated that mechanical recycling appears to be the most desirable method for the management of PET waste, as compared to chemical recycling and incineration [12,13,14]. The chemical or tertiary recycling method involves depolymerization of the PET polymer by chemical agents (chemolysis) or temperature (pyrolysis) to obtain its constituent monomers, that is, monoethylene glycol (MEG) and terephthalic acid (TA), or their derivatives, which can be used for new polymerization processes [15,16]. In addition, the char from pyrolysis of washed PET wastes can successfully replace up to 50 wt% the epoxy resin used in the production of thermosets [17]. In mechanical or secondary recycling, PET waste is subjected to mechanical processes including shredding, grinding, melting, and, when necessary, as in the case of contaminated articles, is combined with washing and/or drying [18]. In this process, the polymers stay intact, which permits multiple reuses in the same or similar products. However, mechanical recycling is currently the preferred option for monomaterial packaging since it has the advantages of simplicity and low cost, requires little investment, uses established equipment, and has little adverse environmental impact. Despite these advantages, mechanical recycling of PET is difficult due to complexity and waste contamination [12,19,20]. Another issue observed during the mechanical recycling of PET by “fusion reprocessing” is that the polymer is habitually subjected to chemical, mechanical, thermal, and oxidative degradation, which decreases the molar mass and, finally, causes the deterioration of the performance and transparency of PET articles [21,22].

Different studies have conventionally supported the notion that the use of recycled polymer streams in a mixture with virgin polymer of the same nature is a good solution for improving the properties of recycled polymer materials and achieving mechanical recycling. Elamri et al. [23] investigated fibers from blends of recycled polyethylene terephthalate (r-PET) and virgin PET. An improvement in the melt processing of r-PET and its fibers, with similar mechanical characteristics to those obtained from virgin PET, was reported. In another study, Scarfato and La Mantia [24] studied recycled and virgin mixtures of polyamide 6 (PA6), showing mechanical and rheological properties similar to those of the virgin polyamide. Moreover, Elamri et al. [25] also investigated blends of recycled and virgin high-density polyethylene (HDPE), reporting a predictable linear behavior in the mechanical properties as a function of the recycled content. A novel, plausible solution to increase the properties of r-PET during melt reprocessing is the use of additives such as chain extenders [26,27,28,29]. This option represents a more economical and attractive strategy than chemical recycling processes for monomaterial packaging since these additives can be used during reprocessing cycles by extrusion or injection molding [26,27]. Chain extenders are additives containing at least two functional groups that are capable of reacting with the end groups of different macromolecular fragments, thereby creating new covalent bonds. During melt reprocessing, these additives are able to “reconnect” the previously broken polymer chains, leading to a polymer with higher molecular weight (M_W_) and restored properties [27,28].

The use of chain extenders during PET reprocessing has been widely investigated. Some studies have reported the use of pyromellitic dianhydride (PMDA) [30,31], organic phosphites [32,33], bis-oxazolines [34,35,36], bis-anhydrides [30,37], diisocyanates [29,34,38], diepoxides [34], epoxy-based oligomers [34,39], or oligomeric polyisocyanates [40]. However, new commercial chain extenders based on random copolymers of poly(styrene-*co*-glycidyl methacrylate) (PS-*co*-GMA) have recently arisen. In particular, the glycidyl methacrylate (GMA) multi-functionality has excellent affinity with condensation polymers, not only PET, but also polybutylene terephthalate (PBT) or polylactide (PLA). It performs by connecting the polymer chains through a chemical reaction of the GMA groups with the hydroxyl (–OH), carboxyl (–COOH), and amine (–NH_2_) terminal groups of the polycondensation polymers. This process can result in a branched structure that also offers higher melt strength for optimal processing conditions. Although some other block copolymers based on GMA groups have been previously used as a chain extenders in other types of polymer blends, such as poly(trimethylene terephthalate) (PTT)/polystyrene (PS) [41] or PET/polypropylene (PP) [42], their use in PET systems remains almost unexplored. Only Benvenuta et al. [43] recently synthesized reactive tri-block copolymers of styrene glycidyl methacrylate (SGMA) and butyl acrylate (BA) (SGMA-*co*-BA-*co*-SGMA) by reversible addition-fragmentation chain transfer (RAFT) and used them as chain extenders of r-PET. These additives turned out to be very effective at increasing the molar mass and intrinsic viscosity, diminishing the melt flow, and improving the melt elasticity and processability of r-PET.

Despite the large amount of work that has been undertaken on the recycling of PET and r-PET blends, those based on bio-PET are barely beginning to be studied. Furthermore, while the price of virgin PET remains stable [44], the use of r-PET is significantly increasing, and thus, novel and more sustainable technologies for PET recycling could encourage more competitive prices in the industry. In the present work, the properties of bio-PET and pre-consumer (uncontaminated) r-PET blends were analyzed to ascertain the potential for mechanical recycling of the next generation of PET materials. To this end, different amounts of r-PET were melt-mixed with bio-PET in a twin-screw industrial extruder and then shaped into pieces by injection molding. During melt compounding, PS-*co*-GMA was added and the effect of the random copolymer on bio-PET/r-PET blends was assessed through a detailed characterization, including measurements of the mechanical, thermal, and thermomechanical properties as well as the rheological behavior.

## 2. Materials and Methods

### 2.1. Materials

Bio-PET, commercial grade BioPET 001, was obtained from NaturePlast (Ifs, France). According to the manufacturer, this grade is produced from up to 30 wt% renewable materials. It has a melting temperature (T_m_) between 240–260 °C, a true density of 1.3–1.4 g·cm^−3^, and an intrinsic viscosity between 75–79 mL·g^−1^ while it is supplied with a water content of less than 0.4 wt%. Further details can be found elsewhere [45]. r-PET was obtained from remnants of the injection blowing process of contaminant-free food-use bottles supplied by the local company Plásticos Guadalaviar S.A. (Beniparell, Spain). The pre-consumer PET waste was shredded and supplied in the form of flakes. PS-*co*-GMA was provided by Polyscope Polymers B.V. (Geleen, The Netherlands) as Xibond^TM^ 920 in the form of pellets. It has a mass average M_W_ of 50,000 g·mol^−1^ and a glass transition temperature (T_g_) of 95 °C. The manufacturer recommends a dosage level of 0.1–5 wt% while not exceeding 330 °C to avoid thermal degradation.

### 2.2. Preparation of the Bio-PET/r-PET Blends

Both bio-PET and r-PET were dried at 60 °C for 72 h, while PS-*co*-GMA was dried at 90 °C for 3 h. All materials were dried separately to avoid premature cross-linking and then premixed in a zipper bag. Table 1 summarizes the coding and compositions of the prepared samples.

The compounding process was carried out in a twin-screw extruder from Construcciones Mecánicas Dupra, S.L. (Alicante, Spain) equipped with a screw diameter of 25 mm and a length-to-diameter (L/D) ratio of 24. The rotating speed was set to 25 rpm and the temperature profile from the feeding to the die was: 240 °C (hopper)–245 °C–250 °C–255 °C (nozzle). The strands were cooled in air and granulated in pellets in an air-knife unit. The resultant pellets were dried at 60 °C for 72 h to remove moisture since PET has a high sensitivity to hydrolysis.

Standard pieces for characterization were obtained from the compounded pellets by injection molding in a Meteor 270/75 from Mateu&Solé (Barcelona, Spain). The temperature profile in the injection machine was programmed to 250 °C (hopper)–255 °C–255 °C–260 °C (injection nozzle). The resultant pieces were finally annealed at 60 °C for 72 h to further develop crystallinity, improve their dimensional stability, and remove any residual moisture.

### 2.3. Mechanical Characterization

Tensile properties were obtained in a universal test machine ELIB-50 from S.A.E. Ibertest (Madrid, Spain) following ISO 527-1:2012. The selected cross-head speed was 5 mm·min^−1^ and the load cell was 5 kN. As recommended by the standard, the tensile modulus (E_t_), maximum tensile strength at break (σ_max_), and elongation at break (ε_b_) values were determined. Shore D hardness was obtained in a 676-D durometer from Instruments J. Bot S.A. (Barcelona, Spain) as indicated in ISO 868:2003. The impact strength was obtained in a Charpy’s pendulum of 1 J from Metrotec (San Sebastián, Spain) on V-notched samples with a radius notch of 0.25 mm according to ISO 179-1:2010. All the mechanical tests were performed under controlled conditions of 25 °C and 40% relative humidity (RH). At least 6 samples of each material were evaluated.

### 2.4. Color Measurements

Changes in color were measured in a colorimetric spectrophotometer ColorFlex from Hunterlab (Reston, VA, USA). The selected color space was the chromatic model L* a* b* or CIELab (spherical color space). L* stands for the luminance, where L* = 0 represents dark and L* = 100 indicates clarity or lightness. The a*b* pair represents the chromaticity coordinate, where a* > 0 is red, a* < 0 is green, b* > 0 is yellow, and b* < 0 is blue. The L*a*b* coordinate values were obtained on five different samples and the color difference (ΔE*) was calculated using the following Equation (1):

(1) $$∆E^{*}={\left[{\left(∆L^{*}\right)}^{2}+{\left(∆a^{*}\right)}^{2}+{\left(∆b^{*}\right)}^{2}\right]}^{0.5}$$

where ∆L*, ∆a*, and ∆b* correspond to the differences between the color parameters of the tested pieces and the values of the neat bio-PET (B100) piece. Color change was evaluated as follows: Unnoticeable (ΔE* < 1), only an experienced observer can notice the difference (ΔE* ≥ 1 and < 2), an unexperienced observer notices the difference (ΔE* ≥ 2 and < 3.5), clear noticeable difference (∆E* ≥ 3.5 and < 5), and the observer notices different colors (ΔE* ≥ 5) [46].

### 2.5. Microscopy

The fracture surfaces of the injection-molded samples after the impact test were covered with a thin metal layer to provide the conducting properties for analysis by field emission scanning electron microscopy (FESEM). The sputtering process was carried out in a cathodic sputter-coater Emitech SC7620 from Quorum Technologies LTD (East Sussex, UK). Subsequently, the morphologies were observed in a CARL ZEISS Ultra-55 FESEM microscope from Oxford Instruments (Abingdon, UK). The acceleration voltage was set to 2.0 kV.

### 2.6. Thermal Characterization

Thermal characterization was carried out on a differential scanning calorimeter DSC-821 from Mettler-Toledo Inc (Schwarzenbach, Switzerland). Samples with a mean weight of 6.1 ± 1.2 mg were placed in aluminum pans and subjected to a dynamic thermal program in three stages: initial heating from 30 °C to 280 °C, cooling to 0 °C, and a second heating to 350 °C. The heating/cooling rates were set at 10 °C ·min^−1^ for all three stages, and the atmosphere was air at a flow-rate of 66 mL·min^−1^. The DSC runs were obtained in triplicate to obtain reliable results. The values of T_g_, cold crystallization temperature (T_cc_), T_m_, as well as the enthalpies corresponding to the melting process (ΔH_m_) and the cold crystallization process (ΔH_cc_) were obtained from the second heating step, whereas the temperature of crystallization (T_c_) and enthalpy of the crystallization process (ΔH_c_) were obtained from the cooling step. The degree of crystallinity (χ_c_) was calculated using Equation (2):

(2) $${\%\chi }_{c}=\frac{\left({\Delta H}_{m}-{\Delta H}_{\mathrm{cc}}\right)}{{\Delta H}_{100\%}\cdot W_{p}}\cdot 100$$

where W_p_ stands for the weight fraction of PET (%) in the sample and ΔH_100%_ is the melting enthalpy of a theoretic 100% crystalline PET, that is, 140 J·g^−1^ [47,48].

Thermal degradation was evaluated by thermogravimetry (TGA) in a TGA/SDTA851 thermobalance from Mettler-Toledo Inc (Schwarzenbach, Switzerland). Samples with an average weight of 5–7 mg were subjected to a temperature sweep from 30 °C to 700 °C at a constant heating rate of 20 °C·min^−1^ in air atmosphere with a flow-rate of 50 mL·min^−1^. The onset degradation temperature, which was assumed at a weight loss of 5 wt% (T_5%_), and the maximum degradation rate temperature (T_deg_) were obtained from the TGA curves. All thermal tests were run in triplicate.

### 2.7. Thermomechanical Characterization

Dynamical mechanical thermal analysis (DMTA) was conducted in an oscillatory rheometer AR-G2 from TA Instruments (New Castle, DE, USA), equipped with a special clamp system for solid samples working in a combination of shear-torsion stresses. The maximum shear deformation (%γ) was set to 0.1% at a frequency of 1 Hz. The thermal sweep was from 20 °C to 160 °C at a heating rate of 5 °C min^−1^. Thermomechanical analysis (TMA) was carried out in a Q-400 thermoanalyzer, also from TA Instruments. A dynamic thermal sweep from 0 °C to 140 °C at a constant heating rate of 2 °C·min^−1^ with an applied load of 20 mN was performed. All thermomechanical tests were also done in triplicate.

## 3. Results and Discussion

### 3.1. Mechanical Properties of the Bio-PET/r-PET Blends

Table 2 shows the mechanical properties in terms of E_t_, σ_max_, ε_b_, Shore D hardness, and impact strength for bio-PET, r-PET, and their blends. One can observe that neat bio-PET resulted in elastic and flexible pieces, having E_t_ and σ_max_ values of approximately 600 MPa and 57 MPa, respectively, whereas ε_b_ nearly reached a value 495%. Shore D hardness and impact strength values were 92.3 kJ·m^−2^ and 2.87 kJ·m^−2^, respectively. These mechanical properties were similar to those reported in our earlier research work [45], though the present pieces were slightly less rigid and more flexible. The performance differences could be related to variations in the moisture content since the PET polyester can easily absorb water due to the high hygroscopicity that plasticizes it [49]. In contrast, neat r-PET produced pieces with a similar mechanical strength but a significantly lower mechanical ductility and toughness. In particular, ε_b_ was 23.1%, while the impact strength was 0.82 kJ·m^−2^, that is, a respective 21- and 3.5-fold reduction in comparison with virgin bio-PET. This ductility impairment has been associated with the decrease in the PET’s M_W_, mainly caused by a phenomenon of chain scission [50,51]. Therefore, as expected, increasing the amount of r-PET in the blend induced a progressive reduction in the ductile performance of the pieces, reaching ε_b_ values in the 10–11% range for the highest contents. The incorporation of r-PET in contents of 30 wt% and 45 wt%, however, increased the E value by approximately 40%. This mechanical behavior has been previously ascribed by Mancini and Zanin to an increase in crystallinity [50] but it can also be associated with the attained reduction in ductility.

**Figure 1:**
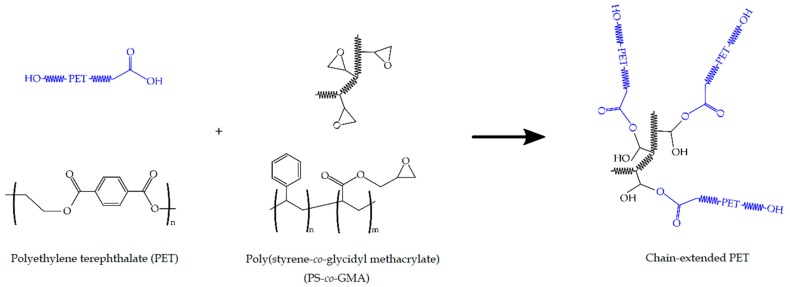
Proposed mechanism of chain extension of polyethylene terephthalate (PET) with poly(styrene-*co*-glycidyl methacrylate) (PS-*co*-GMA).

The incorporation of 1 phr of PS-*co*-GMA into the blend system had a slight influence on the mechanical performance of the bio-PET/r-PET pieces. However, the use of contents of 3 phr and 5 phr successfully yielded a significant increase in the ductility and toughness in the r-PET/bio-PET pieces. In particular, for the blend piece containing 45 wt% r-PET, ε_b_ increased from 10.8% to 312.9% and 378.8% for the same pieces that were melt-processed with 3 phr and 5 phr of PS-*co*-GMA, respectively. The same trend was observed for the impact strength values, increasing from 1.84 kJ·m^−2^ to 2.43 kJ·m^−2^ and 2.52 kJ·m^−2^, respectively. This improved ductility can be ascribed to the chain extension mechanism of PET achieved during extrusion by the presence of the PS-*co*-GMA. This reactive copolymer contains multiple GMA groups that can react with the –OH and –COOH terminal groups of both bio-PET and r-PET. As a result of the so-called reactive extrusion process, a macromolecule of higher M_W_ based on a linear chain-extended, branched, or even cross-linked structure is formed. The proposed chain extension mechanism is shown in Figure 1. Thus, the resultant material shows a macromolecular structure with a higher degree of entanglements to resist mechanical deformation. A similar mechanical improvement was observed, for instance, by the addition of diisocyanates, in which the ε_b_ value notably increased from approximately 5% to 300% [34]. Similarly, maleinized hemp seed oil (MHO) and acrylated epoxidized soybean oil (AESO) have been used to increase the mechanical performance of PLA materials [52].

### 3.2. Morphologgy and Appareance of the Bio-PET/r-PET Blends

**Figure 2:**
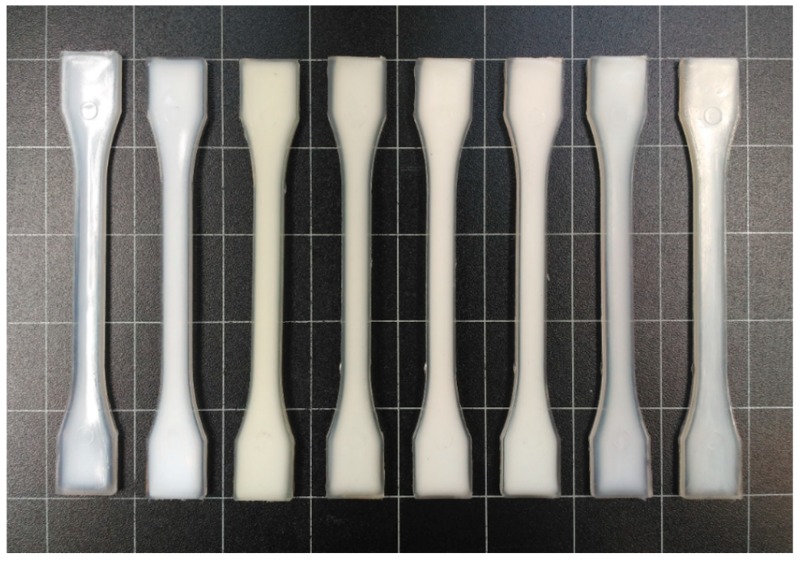
Visual aspect of the injection-molded pieces of the partially bio-based and recycled polyethylene terephthalate (bio-PET/r-PET) blends corresponding, from left to right, to: B100, R100, B85-R15, B70-R30, B55-R45, B55-R45-X1, B55-R45-X3, and B55-R45-X5.

Figure 2 shows the appearance of the injection-molded neat bio-PET, r-PET, and their blends. Color changes of the bio-PET/r-PET pieces were quantified by the L*a*b* coordinates and are reported in Table 3. PET is a semi-crystalline polyester that can be manufactured during injection molding into articles of different transparencies by selecting the appropriate cooling conditions, being very transparent when full amorphous or opaque when it is highly crystallized [53]. The neat bio-PET piece presented a natural bright color, but it was opaque, indicating that the biopolymer developed a certain crystallinity during cooling in the injection mold and in the subsequent annealing. The r-PET pieces were also opaque, but slightly grey and less bright. One can observe that the bio-PET pieces developed a yellowish color (b* > 0) as the weight percentage of r-PET increased, being also opaque. This effect has been described by Torres et al. [29], who attributed it to the presence of spherulitic crystallization as a result of the addition of r-PET. One can also observe that the introduction of PS-*co*-GMA reduced the yellow tonality in the pieces, though the effect of the random copolymer on their visual appearance was relatively low. Therefore, although a different color was noticed (ΔE* ≥ 5) in all the pieces, the color differences were significantly less noticeable after the incorporation of PS-*co*-GMA.

**Figure 3:**
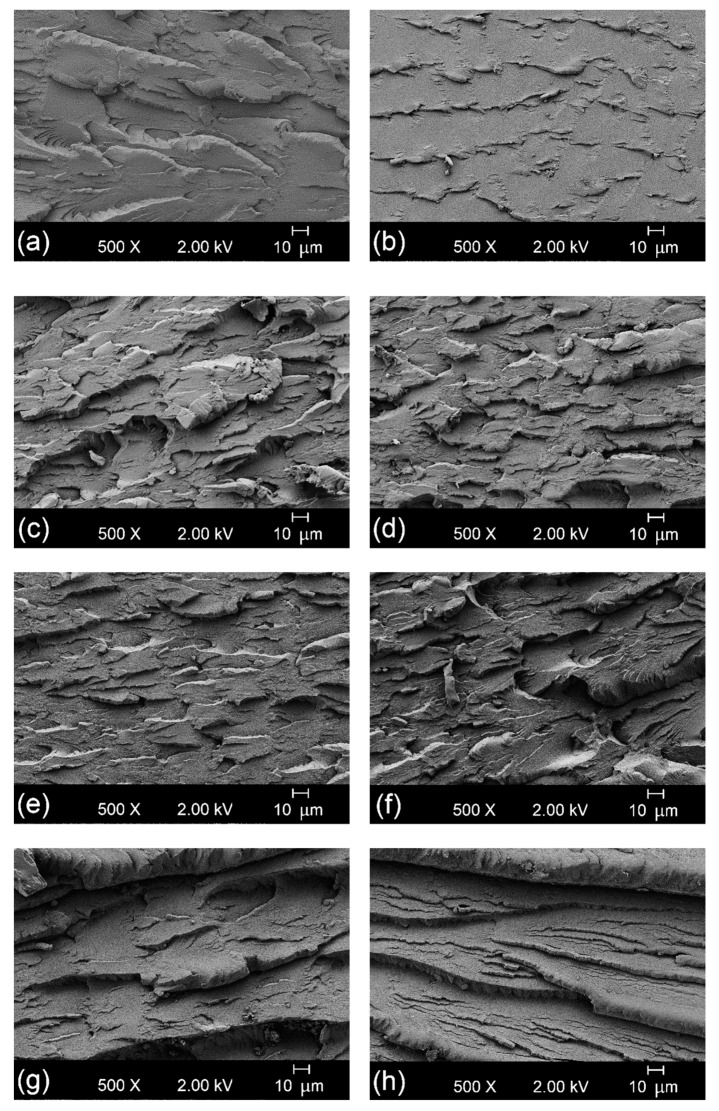
Field emission scanning electron microscopy (FESEM) images, taken at 500×, corresponding to fracture surfaces of the injection-molded pieces of the partially bio-based and recycled polyethylene terephthalate (bio-PET/r-PET) blends: (**a**) B100; (**b**) R100; (**c**) B85-R15; (**d**) B70-R30; (**e**) B55-R45; (**f**) B55-R45-X1; (**g**) B55-R45-X3; (**h**) B55-R45-X5. Scale markers of 10 µm.

Figure 3 presents the FESEM images corresponding to fracture surfaces of the injection-molded pieces after the impact tests. All morphologies showed the formation of microcracks that were responsible for the final fracture of the material. Figure 3a, which corresponds to the FESEM micrograph of the neat bio-PET piece, shows that the size of the cracking steps was larger with somewhat more rounded edges compared with those of the r-PET piece shown in Figure 3b. This morphological change is due to more energy being required during the breakage process so that the fracture produced a rougher surface. In both cases, the cracking steps were uniform. In contrast, the FESEM micrographs corresponding to the bio-PET/r-PET blends, shown in Figure 3c–e, yielded very heterogeneous fracture surfaces. This type of fracture can be mainly ascribed to the hardness increase of the injection-molded piece [54]. Furthermore, although the morphologies revealed the presence of a single phase, this type of surface can also be ascribed to the lack of chemical interaction between the matrix, that is, bio-PET, and the dispersed domains of r-PET. One can observe in Figure 3f that a similar morphology was obtained when the polyester blend was melt-processed with 1 phr of PS-*co*-GMA. However, as seen in Figure 3g,h, the addition of the 3 phr and 5 phr of PS-*co*-GMA produced pieces with fracture surfaces very similar to those observed for neat bio-PET. In particular, one can observe that larger cracks with rounded edges were produced due to the aforementioned increase in toughness. Therefore, the fracture surfaces correlated well with the mechanical strength and impact strength performance attained during the mechanical analysis. A similar morphology was reported by Badia et al. [55], who showed a smoother surface loss in the surface fractures of virgin PET after the addition of r-PET. This behavior was also noted in other recycled polymers [56,57].

### 3.3. Thermal Characterization of the Bio-PET/r-PET Blends

**Figure 4:**
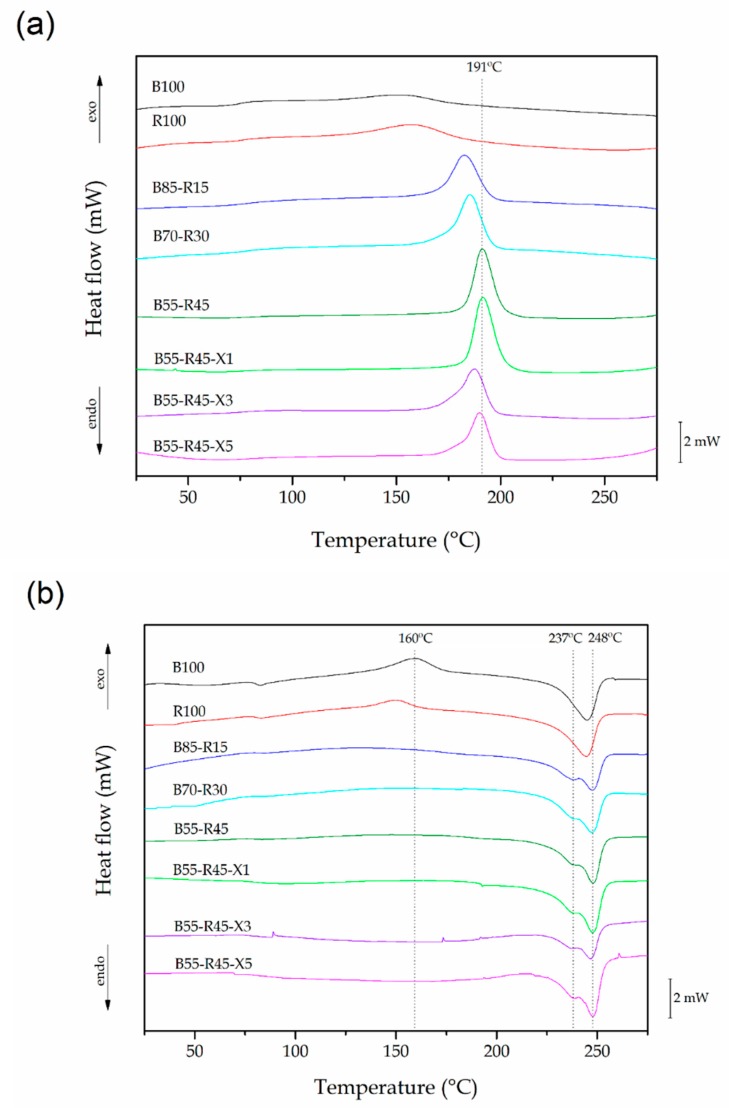
Differential scanning calorimetry (DSC) curves during cooling (**a**) and second heating (**b**) of the injection-molded pieces of the partially bio-based and recycled polyethylene terephthalate (bio-PET/r-PET) blends.

The thermal transitions and degree of crystallinity are parameters of great technological importance that reflect the chemical structure of the polymer and they can be correlated with its mechanical properties [58]. Figure 4 shows a comparative plot of the DSC thermograms of the bio-PET/r-PET blends during the cooling step (Figure 4a) and second heating step (Figure 4b). The main thermal properties obtained from the DSC analysis are summarized in Table 4. In relation to the bio-PET sample, the glass transition region was seen as a step in the baseline located around 82 °C in the heating thermogram. Then, one can observe in the cooling thermogram that the biopolyester did not crystallize from the melt but developed a cold crystallization process during heating in the thermal range between 140 °C and 180 °C, showing a maximum exothermic peak, the so-called T_cc_, at 161 °C. Finally, shown in the heating thermogram, the endothermic peak between 220 °C and 260 °C corresponds to the melting process of the total crystalline fraction in the biopolyester, showing a T_m_ value of nearly 245 °C. The thermal parameters attained for bio-PET were very similar to those obtained in our previous work [45]. A similar thermal profile was also observed for r-PET, with the most significant difference being the lower T_cc_ value observed, which was located at approximately 150 °C. This observed value can be ascribed to the lower M_W_ of r-PET, which compromises shorter chains that can more easily cold crystallize due to the fact that the polyester was subjected to a second round of processing [59].

Interestingly, the bio-PET/r-PET blends showed a different thermal profile during DSC analysis. One can observe that the polyester blends crystallized in all cases during cooling, showing values of T_c_ in the 180–190 °C range. This observation suggests that crystallization was thermodynamically favored in the PET blends. This phenomenon can be ascribed to the potential role of r-PET as a nucleating agent in bio-PET. In this regard, the nucleating effect of r-PET has been previously related to the presence of impurities [29]. Therefore, the spherulitic crystallization of r-PET occurs at lower temperatures and the crystals formed can thereafter promote the homogenous crystallization of PET chains at higher temperatures. This effect was further confirmed by the increase in the T_c_ values and the higher intensity of the exothermic peaks observed during crystallization from the melt with higher r-PET contents. Thus, χ_c_ value increased from 10.6% in the neat bio-PET sample to 29% for the bio-PET blend containing 15 wt% of r-PET, whereas these values reached 32.3% and 37% for the blends containing 30 wt% and 45 wt% of r-PET, respectively. It is also worth mentioning that while the melting process of the neat bio-PET sample occurred in a single melt peak, all the bio-PET/r-PET blends showed two overlapping peaks during melting. This double-melting peak phenomenon can be explained by the presence of dominant and subsidiary crystals [60]. The two melting peaks (T_m1_ and T_m2_) were observed at approximately 238 and 248 °C, with the latter temperature being assigned to primary folded chain crystals with a higher melting point than those observed for the neat bio-PET. This observation further confirms that the presence of r-PET in bio-PET favors the formation of more perfect crystalline structures, which were obtained from the melt of imperfect structures or crystals with lower lamellae thicknesses [61]. Similar results were observed by Spinacé and De Paoli [62], who indicated that the chain scission, occurring during the processing of PET, improved chain packing, increasing the crystallite size and consequently shifting T_c_ and T_m_ to higher values.

The incorporation of PS-*co*-GMA barely modified the T_g_, T_m1_, and T_m2_ values, though the use of contents of 3 phr and 5 phr decreased the crystallinity to 17.7% and 25.9%, respectively. The reactive random copolymer also caused a slight reduction in the T_c_ values, showing values around 185 °C, indicating that the structural disorder introduced by the chain extender potentially hindered both nucleation and crystallization. These observations agree with the aforementioned assumption that the induced M_W_ increase could decrease the molecular mobility, resulting in a low crystallization rate and lower crystallinities [63]. Moreover, Kiliaris et al. [64] also concluded that the ability of the PET chains to crystallize in folded lamellae was limited after chain extension, which led to the formation of smaller and less perfect crystallites. Furthermore, if one considers branching as the prevailing chain-extension reaction, the presence of the resultant branching points could effectively hinder chain symmetry, restricting the segment movements and yielding crystal defects.

**Figure 5:**
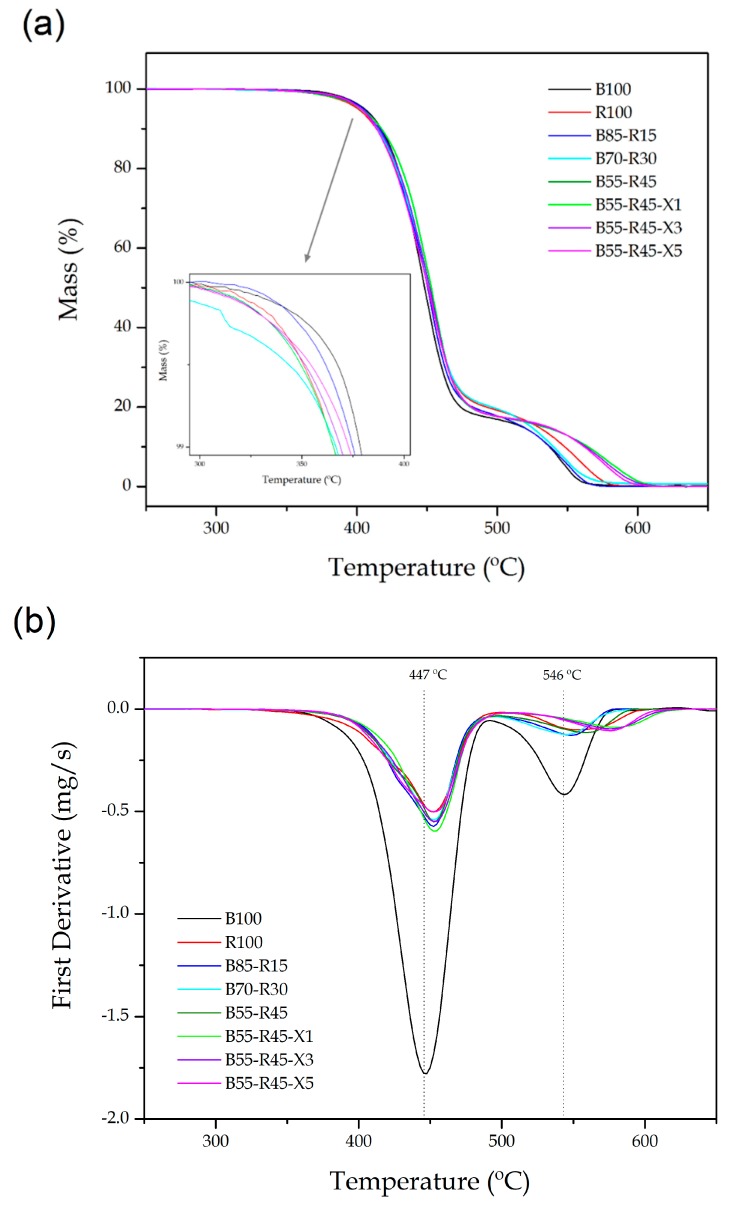
(**a**) Thermogravimetric analysis (TGA) and (**b**) first derivative thermogravimetric (DTG) curves of the injection-molded pieces of the partially bio-based and recycled polyethylene terephthalate (bio-PET/r-PET) blends.

One of the main problems related to the mechanical recycling of thermoplastic materials is the loss of thermal stability. Figure 5 gathers the TGA curves (Figure 5a) and the first derivatives of the curves (DTG) (Figure 5b) of the bio-PET/r-PET blends, while Table 5 presents the thermal values obtained from the TGA curves. It can be observed that bio-PET degradation occurred in two stages, as previously reported by Vannier et al. [65]. The first stage, occurring from 350 °C to 460 °C, corresponds to the main degradation of the biopolymer backbone and the formation of char with a mass loss of ~83%. The second step, which was associated with a loss of nearly the totality of the remaining biopolyester, was related to the thermo-oxidative degradation of the char. This occurred from 490 °C to 560 °C. It has also been reported that the decomposition mechanism of PET consists of an hererolytic scission via a six-membered ring intermediate, where the hydrogen from a β-carbon to the ester group is transferred to the ester carbonyl, followed by scission at the ester links [66]. Dziȩcioł and Trzeszczyński [67,68] also reported that the degradation of PET leads to the formation of acetaldehyde, oligomers with terminal carboxyl groups, CO, and CO_2_, among other compounds. In all cases, the bio-PET/r-PET samples resulted in a residual mass of 1–2 wt% at 700 °C.

One can also observe that the thermal degradation profile of r-PET and the bio-PET/r-PET blends was similar to that of the neat bio-PET. The addition of PS-*co*-GMA yielded an increase in the onset temperature of degradation that was measured as the degradation temperature at 5% of mass loss, that is, T_5%_. In particular, the beginning of the thermal degradation was delayed from approximately 393 °C for the blends containing 30 wt% and 45 wt% of r-PET to nearly 403 °C. The thermal degradation peaks (T_deg1_ and T_deg2_), determined when the maximal degradation rates were produced, remained nearly constant during the first and main mass loss, while they slightly increased during the second one in the PET blends processed without PS-*co*-GMA. In particular, the T_deg2_ values were increased by up to nearly 19 °C, which suggests a delay in the thermo-oxidative degradation of the char. In any case, the influence of the reactive copolymer on the thermal degradation of the bio-PET/r-PET blends was relatively low, since the thermal stability of the polyester blends was already high.

### 3.4. Thermomechanical Properties of the Bio-PET/r-PET Blends

**Figure 6:**
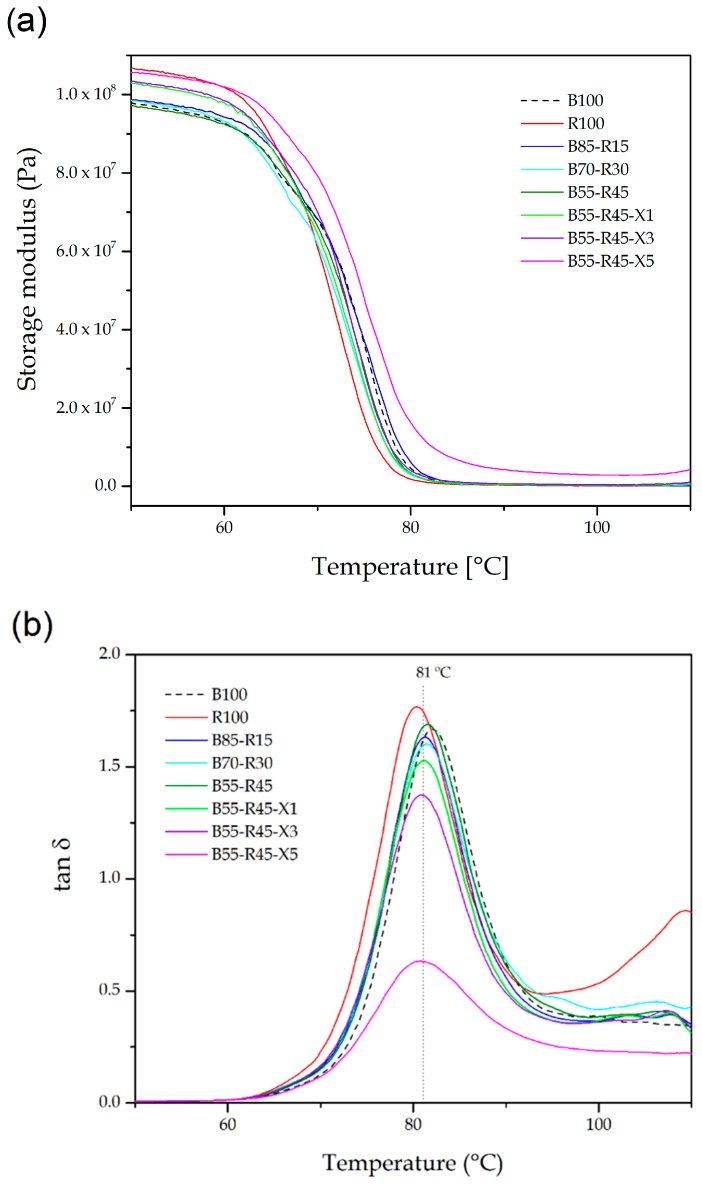
(**a**) Storage modulus and (**b**) damping factor (*tan δ*) of the injection-molded pieces of the partially bio-based and recycled polyethylene terephthalate (bio-PET/r-PET) blends.

The prepared bio-PET/r-PET blends and the effect of the reactive random copolymer on these polyester blends were analyzed in terms of their thermomechanical properties by means of DMTA and TMA. Figure 6 shows the DMTA curves of the bio-PET/r-PET blend pieces. Figure 6a shows the storage modulus as a function of temperature. The evolution of the storage modulus of the bio-PET was characterized by a single thermal transition with a temperature increase in the 50–130 °C range. Up to approximately 70 °C, the variation in the storage modulus values was nearly negligible since the biopolyester was in a glassy state. At room temperature, all the pieces showed similar storage modulus values, being around 1 GPa and slightly lower for the neat bio-PET due to its reduced crystallinity. Thereafter, one can observe a remarkable decrease in the storage modulus values in the temperature range from 70 °C to 85 °C. This process notably involved a decrease of more than two orders of magnitude in the storage modulus, which is representative for the glass-to-rubber transition of bio-PET. At higher temperatures, the variation in the storage modulus was nearly negligible, indicating that the biopolyester reached its rubber state and that further temperature increase did not affect the mechanical properties. Table 6 presents the values of the storage modulus obtained at 60 °C and 100 °C since these temperatures are representative of the mechanical rigidity of the bio-PET pieces before and after the glass transition region of bio-PET. While at 60 °C, the value of the storage modulus of the neat bio-PET piece was nearly 900 MPa, this value was reduced to approximately 3 MPa at 100 °C. It can also be observed that the r-PET piece and all the bio-PET/r-PET pieces showed similar thermochemical performance with slightly higher storage modulus values. The incorporation of PS-*co*-GMA led to an increase in the storage modulus values, in both the glassy and the rubber states. The highest enhancement was obtained for the bio-PET/r-PET blend piece processed with 5 phr of PS-*co*-GMA, in which it reached values of close to 1.2 GPa and 30 MPa at 60 °C and 100 °C, respectively. This thermomechanical increase can be ascribed to the aforementioned formation of a chain-extended macromolecular structure with higher M_W_ that showed increased resistance to flow during the application of external forces as a result of the large number of entanglements associated with the long chains or branches [69].

Figure 6b shows the evolution of the damping factor (*tan δ*) as a function of temperature. The maximum peak of the *tan δ* curves corresponds to alpha (α)-transition of bio-PET, which is related to its T_g_. These values are also given in Table 6, where it can be seen that for neat bio-PET, a T_g_ value of approximately 81 °C was observed, being nearly the same as that obtained above by DSC. A slight lower value of T_g_, that is, ~80 °C, was observed for r-PET, with this potentially being related to its lower chain lengths and/or the presence of plasticizers. The bio-PET/r-PET blends showed intermediate values of T_g_, ranging between 80 °C and 81 °C, being also similar to those obtained by DSC. The biopolyester blends melt-processed with PS-*co*-GMA showed nearly the same T_g_ values but with a remarkable reduction in the α-peak intensities. This observation supports the higher crystallinity and the large number of entanglements achieved in the polyester blends based on the fact that the amorphous phase content was reduced due to the presence of r-PET, which partially suppressed the relaxation of the bio-PET chains and lowered the number of molecules undergoing α-transition [70].

Finally, the dimensional stability the bio-PET/r-PET blend pieces was determined by measuring the CLTE values below and above the glass transition region. As also shown in Table 6, below T_g_, bio-PET showed a lower value than r-PET, that is, 78.9 µm·m^−1^·°C^−1^ versus 90.9 µm·m^−1^·°C^−1^. This thermomechanical difference can be ascribed to the lower M_W_ attained in the recycled polyester due to the aforementioned phenomenon of chain scission during reprocessing. Then, the bio-PET/r-PET blends presented intermediate values according to their r-PET content. The incorporation of PS-*co*-GMA induced a reduction in the CLTE values, increasing with the reactive random copolyester content. In particular, a value of 70.6 µm·m^−1^·°C^−1^ was reached for the bio-PET/r-PET blend containing 5 phr PS-*co*-GMA. This observation further supports the formation of a branched and larger macromolecule that reduced the effect of temperature on the dimensional stability, which is also in agreement with the DMTA results. A similar trend with more significant differences was observed for the CLTE values measured above T_g_.

## 4. Conclusions

PS-*co*-GMA, a multi-functional random copolymer, was melt-mixed with bio-PET/r-PET blends at contents of 1–5 phr of polyester blend and shaped into pieces by injection molding. The resultant pieces were characterized to ascertain the potential use of PS-*co*-GMA as a chain extender for the mechanical recycling of these polyester blends. The results showed that while the incorporation of 1 phr of PS-*co*-GMA had a slight influence on the mechanical and thermal performance of the bio-PET/r-PET blend pieces, the contents of 3 phr and 5 phr successfully yielded a significant increase in their ductility and toughness. In particular, ε_b_ increased from 10.8%, for the blend piece containing 45 wt% of r-PET, to 312.9% and 378.8%, for the same pieces that were melt-processed with 3 phr and 5 phr of PS-*co*-GMA, respectively. In addition, the impact strength values increased from 1.84 kJ·m^−2^ to 2.43 kJ·m^−2^ and 2.52 kJ·m^−2^, respectively. This mechanical improvement was ascribed to the chain extension mechanism of PET by reactive extrusion due to the reaction of the multiple GMA groups that are present in PS-*co*-GMA with the –OH and –COOH terminal groups of both bio-PET and r-PET. Furthermore, PS-*co*-GMA reduced the crystallinity of the PET blends, suggesting that the structural disorder introduced by the chain extender potentially hindered both the nucleation and crystallization of r-PET on the bio-PET chains, contributing to increase material flexibility. In addition, the influence of the reactive copolymer on the thermal stability of the bio-PET/r-PET blends was low, since the blends were already thermally stable up to nearly 400 °C. The thermomechanical properties of the bio-PET/r-PET blends also improved after the addition of PS-*co*-GMA due to the formation of a branched and larger macromolecule.

Bio-PET can be effectively mixed with its recycled petrochemical counterpart, that is, r-PET, and then mechanically recycled in existing recycling facilities by means of reactive extrusion with PS-*co*-GMA. Therefore, the original biopolymer properties can be successfully restored and the ultimate performance of bio-PET articles would be retained for a given number of reprocessing cycles. This will permit the recovery of upcoming bio-PET streams with current r-PET waste to manufacture the same or similar products. According to this scenario, mechanical recycling for bio-based but non-biodegradable polymers will be appropriate from both an economic and environmental point of view. This will potentially contribute to accelerating the transition of the plastic packaging industry from its traditional linear model to a more valuable and sustainable circular model.

## Figures and Tables

**Table 1:** Code and composition of each sample according to the weight content of partially bio-based (bio-PET) and recycled polyethylene terephthalate (r-PET) in which poly(styrene-*co*-glycidyl methacrylate) (PS-*co*-GMA) was added as parts per hundred resin (phr) of blend.

Sample	bio-PET (wt%)	r-PET (wt%)	PS-*co*-GMA (phr)
B100	100	-	-
R100	-	100	-
B85-R15	85	15	-
B70-R30	70	30	-
B55-R45	55	45	-
B55-R45-X1	55	45	1
B55-R45-X3	55	45	3
B55-R45-X5	55	45	5

**Table 2:** Summary of the mechanical properties of the partially bio-based and recycled polyethylene terephthalate (bio-PET/r-PET) blends in terms of tensile modulus (E_t_), maximum tensile strength (σ_max_), elongation at break (ε_b_), Shore D hardness, and impact strength.

Piece	E_t_ (MPa)	σ_m__ax_ (MPa)	ε_b_ (%)	Shore D Hardness	Impact Strength (kJ·m^−2^)
B100	600.3 ± 19.1	56.8 ± 0.8	494.6 ± 9.7	92.3 ± 0.9	2.87 ± 0.42
R100	558.8 ± 11.7	56.1 ± 0.7	23.1 ± 4.7	79.8 ± 0.8	0.82 ± 0.13
B85-R15	665.4 ± 19.2	56.6 ± 2.7	20.4 ± 4.9	77.8 ± 1.2	1.73 ± 0.06
B70-R30	822.8 ± 25.6	57.5 ± 1.0	10.5 ± 1.1	77.3 ± 1.3	1.84 ± 0.29
B55-R45	820.5 ± 30.2	57.7 ± 2.8	10.8 ± 1.4	76.0 ± 1.4	1.84 ± 0.38
B55-R45-X1	742.9 ± 10.9	58.1 ± 1.1	11.2 ± 0.9	79.8 ± 1.5	1.89 ± 0.24
B55-R45-X3	852.9 ± 21.8	58.3 ± 0.3	312.9 ± 7.3	81.0 ± 1.2	2.43 ± 0.29
B55-R45-X5	847.8 ± 11.7	57.4 ± 0.1	378.8 ± 8.4	82.8 ±1.8	2.52 ± 0.25

**Table 3:** Color coordinates by CIElab color space (L*a*b*) of the partially bio-based and recycled polyethylene terephthalate (bio-PET/r-PET) blend pieces.

Piece	L*	a*	b*	ΔE*
B100	75.67 ± 0.58	−2.72 ± 0.16	−5.27 ± 0.32	-
R100	42.86 ± 0.37	−0.54 ± 0.11	−2.62 ± 0.10	32.99 ± 1.31
B85-R15	68.03 ± 0.44	−3.63 ± 0.07	5.27 ± 0.25	10.84 ± 0.57
B70-R30	64.85 ± 0.48	−2.95 ± 0.14	5.62 ± 0.18	15.35 ± 1.18
B55-R45	63.44 ± 0.96	−2.61 ± 0.14	5.41 ± 0.50	16.24 ± 0.90
B55-R45-X1	69.88 ± 0.52	−1.97 ± 0.17	0.57 ± 0.23	8.26 ± 1.13
B55-R45-X3	72.71 ± 0.86	−2.56 ± 0.13	0.33 ± 0.20	6.34 ± 0.77
B55-R45-X5	73.73 ± 0.98	−2.34 ± 0.17	1.35 ± 0.31	6.91 ± 0.84

**Table 4:** Summary of the main thermal properties of the injection-molded pieces of the partially bio-based and recycled polyethylene terephthalate (bio-PET/r-PET) blends in terms of glass transition temperature (T_g_), cold crystallization temperature (T_cc_), crystallization temperature (T_c_), melting temperature (T_m_), and degree of crystallinity (χ_c_).

Piece	T_g_ (°C)	T_cc_ (°C)	T_c_ (°C)	T_m1_ (°C)	T_m2_ (°C)	χ_c_ (%)
B100	82.0 ± 0.7	150.8 ± 0.4	-	244.9 ± 0.9	-	10.6 ± 0.5
R100	80. 4± 0.8	157.6 ± 0.2	-	244.5 ± 1.1	-	24.2 ± 0.5
B85-R15	81.2 ± 0.6	-	182.7 ± 0.3	238.4 ± 0.8	247.4 ± 1.0	29.0 ± 0.8
B70-R30	81.5 ± 0.8	-	185.1 ± 0.2	238.9 ± 0.7	247.2 ± 0.8	32.3 ± 0.9
B55-R45	82.6 ± 0.9	-	191.2 ± 0.3	237.9 ± 1.0	247.8 ± 1.1	37.0 ± 0.7
B55-R45-X1	81.0 ± 0.7	-	191.4 ± 0.1	238.0 ± 1.1	247.5 ± 0.9	38.1 ± 0.8
B55-R45-X3	80.9 ± 0.8	-	187.4 ± 0.2	236.8 ± 0.8	247.9 ± 0.8	17.7 ± 0.5
B55-R45-X5	80.6 ± 0.7	-	189.8 ± 0.2	237.1 ± 0.7	247.9 ± 1.2	25.9 ± 0.9

**Table 5:** Summary of the main thermal properties of the injection-molded pieces of the partially bio-based and recycled polyethylene terephthalate (bio-PET/r-PET) blends in terms of the degradation temperature at 5% of mass loss (T_5%_), degradation temperature (T_deg_), and residual mass at 700 °C.

Piece	T_5%_ (°C)	T_deg1_ (°C)	T_deg2_ (°C)	Residual Mass (%)
B100	405.4 ± 0.2	446.8 ± 0.1	546.0 ± 0.2	1.26 ± 0.04
R100	400.8 ± 0.4	452.3 ± 0.2	552.7 ± 0.1	1.43 ± 0.07
B85-R15	400.2 ± 0.2	452.2 ± 0.3	549.4 ± 0.2	1.22 ± 0.05
B70-R30	393.2 ± 0.3	452.3 ± 0.2	545.7 ± 0.1	1.64 ± 0.18
B55-R45	393.0 ± 0.2	452.9 ± 0.3	559.5 ± 0.2	2.18 ± 0.05
B55-R45-X1	403.2 ± 0.2	452.9 ± 0.2	578.3 ± 0.2	1.27 ± 0.08
B55-R45-X3	403.1 ± 0.1	452.4 ± 0.1	576.6 ± 0.4	1.73 ± 0.15
B55-R45-X5	403.0 ± 0.1	451.8 ± 0.2	576.1 ± 0.2	2.29 ± 0.06

**Table 6:** Storage modulus measured at 60 °C and 100 °C, glass transition temperature (T_g_), and coefficient of linear thermal expansion (CLTE) of the injection-molded pieces of the partially bio-based and recycled polyethylene terephthalate (bio-PET/r-PET) blends.

Piece	Storage Modulus (MPa)		T_g_ (°C)	CLTE (µm·m^−1^·°C^−1^)	
	60 °C	100 °C		Below T_g_	Above T_g_
B100	927.4 ± 20.5	2.8 ± 0.1	81.0 ± 0.1	78.9 ± 1.9	80.5 ± 1.9
R100	1018.5 ± 20.2	1.7 ± 0.2	80.3 ± 0.1	90.9 ± 1.7	94.9 ± 0.9
B85-R15	943.4 ± 23.2	2.5 ± 0.1	81.2 ± 0.1	80.7 ± 1.1	90.1 ± 1.0
B70-R30	931.6 ± 32.3	2.3 ± 0.1	81.3 ± 0.1	84.1 ± 1.1	103.2 ± 0.2
B55-R45	924.3 ± 13.2	2.6 ± 0.1	81.5 ± 0.1	93.1 ± 1.2	111.8 ± 0.9
B55-R45-X1	977.7 ± 17.3	3.4 ± 0.2	81.1 ± 0.1	90.1 ± 0.5	104.0 ± 1.4
B55-R45-X3	984.4 ± 27.0	4.7 ± 0.2	80.9 ± 0.2	74.9 ± 0.1	101.3 ± 1.1
B55-R45-X5	1019.7 ± 18.4	4.9 ± 0.2	80.8 ± 0.2	70.6 ± 0.3	99.9 ± 1.8
